# Picroside II Improves Severe Acute Pancreatitis-Induced Hepatocellular Injury in Rats by Affecting JAK2/STAT3 Phosphorylation Signaling

**DOI:** 10.1155/2021/9945149

**Published:** 2021-07-27

**Authors:** Xuehua Piao, Xiaodan Sui, Baohai Liu, Tingfang Cui, Zinan Qi

**Affiliations:** ^1^Department of Traditional Chinese Medicine, The First Affiliated Hospital, Jinzhou Medical University, Jinzhou 121001, China; ^2^Department of Hepatology, The Affiliated Hospital of Changchun University of Traditional Chinese Medicine, Changchun 130021, China; ^3^Department of Gastroenterology, The First Affiliated Hospital, Jinzhou Medical University, Jinzhou 121001, China

## Abstract

Picroside II is an important ingredient agent in Traditional Chinese medicine and hoped to reduce hepatocellular injury caused by severe acute pancreatitis (SAP). An SAP-induced hepatocellular injury model was established in rats by using pentobarbital sodium. 27 rats were divided into 3 groups: the sham group (SG), model group (MG), and Picroside groups (PG). SAP-induced hepatocellular injury was assessed using hematoxylin and eosin staining. We measured hepatocellular enzymes (amylase (AMY), alanine aminotransferase (ALT), and aspartate aminotransferase (AST)), oxidative stress factors (superoxidase dismutase (SOD) and malondialdehyde (MDA)), and inflammatory factors (tumor necrosis factor *α* (TNF-*α*), interleukin- (IL-) 6, and IL-10), apoptotic factors (BAX and cleaved caspase 3), and inflammatory signaling (Janus kinase 2 (JAK2)/signal transducer and activator of transcription 3 (STAT3), p-JAK2, and p-STAT3) in hepatocellular tissues. The SAP-induced hepatocellular injury model was successfully established. Picroside II treatment repaired hepatocellular injury by reducing the activities of AMY, ALT, and AST; reducing the levels of MDA, TNF-*α*, IL-1, IL-6, p-JAK2, p-STAT3, BAX, and cleaved caspase 3; and increasing the levels of SOD and IL-10. Picroside II exerted protective function for the SAP-induced hepatocellular injury model. Picroside II improved SAP-induced hepatocellular injury and antioxidant and anti-inflammatory properties by affecting JAK2/STAT3 phosphorylation signaling.

## 1. Introduction

Severe acute pancreatitis (SAP) is a remarkably serious illness in the pancreas, which is associated with numerous tissue failures and high risk of morbidity and mortality [1, 2]. SAP development can straightforwardly induce hepatocellular injury [3, 4]. Furthermore, the hepatic injury cannot only worsen pancreatitis state but also develop into hepatic failure and trigger mortality of SAP patients [5].

Although diagnosis and therapy technology for SAP have improved considerably, no medication is used to treat SAP specifically and an efficient curative drug is still hard to be accessible. Rapid antibiotic treatment is suggested once inflammatory indicators are elevated in severe pancreatitis, to stop the pancreatic infection. Unluckily, there are still persistent controversial questions for antibiotic administration and the subjects who benefit from antibiotic therapy in pancreatitis [6]. Natural products have been considered for the therapy of SPA and its induced organ failures with few side effects. For instance, emodin, a natural anthraquinone compound isolated from the herb *Rheum officinale Baill*, exerts significant anti-inflammatory activities and has been found to be beneficial for the recovery of SAP by affecting via the P2X ligand-gated ion channel 7/NOD-like receptor protein 3 signaling pathway [7]. Picroside II, an active essential obtained from *Picrorhiza kurrooa* [8], Pseudolysimachion rotundum var. subintegrum [9], and *Picrorhiza scrophulariiflora* [10], is undergoing the preclinical study and exhibits dose-dependent protection of the hepatocellular injury [10] and has significant antioxidant and anti-inflammatory properties [11].

In our previous work, we found that Picroside II improved SAP by preventing NF-*κ*B-dependent autophagy [12] or SAP-induced intestinal barrier injury by affecting toll-like receptor 4- (TLR4-) dependent the phosphatidylinositol 3-kinase/protein kinase B/nuclear factor-*κ*B (JAK2/AKT/STAT3) signaling and gut microbiota [13]. However, the effects of Picroside II on SAP-induced hepatocellular injury remain widely unknown. Janus kinase/signal transducers and activators of transcription (JAK/STAT) signaling is one of the main signaling for cytokine signal transduction in hepatocellular injury during SAP [3]. Therefore, in this study, we aimed to explore the related molecular mechanisms by investigating the effects of Picroside II on the related molecules of JAK2/STAT3 signaling in the SAP-induced hepatocellular injury model.

## 2. Materials and Methods

### 2.1. Chemicals

Picroside II (purity > 98%, CAS Number: 39012-20-9) and sodium taurocholate (CAS Number: 145-42-6) were purchased from Aladdin and dissolved in saline solution to a final concentration of 1 mg/mL and 4 mg/mL, respectively. AMS detection kit was purchased from Regen (CAS Number: TE0203). Chemical agents, all other ELISA kits, and antibodies were purchased from Wanleibio (Shenyang, China).

### 2.2. Establishment of the SAP-Induced Hepatocellular Injury Model

All experimental steps were approved by the Animal Research Ethics Committee of Jinzhou Medical University and consistent with Guidelines for the Ethical Review of Laboratory Animal Welfare, People's Republic of China National Standard GB/T 35892-2018 [14]. Twenty-seven male Sprague-Dawley (SD) rats (8 weeks old; weighing 200–220 g) were purchased from the animal center of Jinzhou Medical University according to a previous report [15]. The rats were kept under a Light (L) phase–Dark (D) phase (12 : 12) cycle. Oral administration of 50 mg/kg of metronidazole was performed to reduce the postsurgery pain. All rats were fed a standard rodent diet (from Shanghai Slac Laboratory Animal Co., Ltd.), and AIN93-M diet was recommended by the American Institute of Nutrition. Food and water were made available ad libitum.

All rats were fasted for 12 h before operation, freely drinking water, anesthetized with pentobarbital sodium 40 mg/kg intraperitoneal injection, fixed on the supine position, locally disinfected with 75% alcohol, and covered with sterile perforated towels. A midline abdominal incision was made into the abdominal cavity (the incision was about 2 cm long), and the stomach was stretched to the left. The duodenum is located behind the stomach, and the inside of the duodenal ring is the pancreatic tissue. The operators used the right index finger and thumb to lift most of the duodenum out of the abdomen and found the duodenum and pancreaticobiliary duct of the rat. At the 0.5 cm lower end of the duodenal papilla, the duodenal wall is the avascular area. An intravenous indwelling trocar (24G, 0.7 mm) was used to pierce the intestinal wall and then withdraw the part of the needle core slightly and penetrate the bile pancreatic duct along the direction of the nipple. It could be seen that bile returned in the intravenous indwelling needle. In parallel, the needle was pushed 0.5-1.0 cm anteriorly in the bile-pancreatic duct and fixed. At the same time, the hilar bile duct was clamped with a microvascular clip to prevent the drug from flowing back into the liver and duodenum. At this time, trocar core was completely exited. One mL of syringe containing 4% sodium taurocholate was connected with the end of the cannula, and 1 mL/kg sodium taurocholate was injected retrogradely at a speed of 0.25 mL/min. After 1-2 min, pancreatic congestion, edema, and pancreaticobiliary ducts were seen, and the main pancreatic duct was dilated. After 5 min observation, the vascular clip was removed and the tube was extubated. After confirming that there was no active bleeding in the abdominal cavity, the abdomen was closed in two layers and the wound was covered with a sterile gauze. In the sham operation group (*n* = 9), only the duodenum was turned and the pancreas was touched several times after the abdomen was opened. After the rats were successfully modeled, they were treated with drugs. Among them, the rats in the sham group (*n* = 9) and the model group (*n* = 9) were intraperitoneally injected with 2 mL of normal saline within 5 minutes after successful modeling. According to our previous work [12], SAP rats were administrated with different concentrations of Picroside II (12.5, 25, and 50 mg/kg), and the optimal dose of Picroside II was 25 mg/kg based on the levels of serum amylase and lipase and disease scores. In the treatment group (*n* = 9), 2 mL of 25 mg/kg Picroside II was intraperitoneally injected within 5 minutes after the successful model establishment.

### 2.3. Measurement of Liver Enzyme Activities

The rats were fasted for 12 h before the detection of the live enzymes. The enzymes, amylase (AMY) [16], alanine aminotransferase (ALT) [17, 18], and aspartate aminotransferase (AST) [18] are widely reported biomarkers in liver disease or injury. After 12-hour SAP modelling, one mL blood samples were taken from the post cava of rats via the catheter using sterile syringes, and serum was isolated via centrifugation 3,000 × g at 4°C for 10 min. Serum levels of AMY, ALT, and AST were measured to explore the hepatocellular injury. Serum AMY activity was determined using an AMY kit, and its absorbing values were measured at 660 nm. The serum ALT and AST activities were determined via their kits, and absorbing values were measured at 510 nm.

### 2.4. Animal Grouping

After the SAP-induced hepatocellular injury model was established, 27 animals were thus evenly divided into 3 groups according to different treatments, the sham group (SG), the model group (MG), and Picroside II group (PG). After 6-hour, 12-hour, and 24-hour model establishment, three rats from each group were sacrificed. The serum, liver, and pancreas of rats in each group were taken at 6 h, 12 h, and 24 h after the administration. Three rats were randomly selected from each group at each time point. Among them, some of the liver tissues and pancreas tissues need to be cryopreserved, and some tissues were paraffin-embedded for subsequent testing.

### 2.5. Measurement of Cholestasis

Sodium taurocholate was injected in the common duct, and bile acids would be mediated by sodium taurocholate [19]. Accumulation of bile acids in cholestasis will result in liver inflammation and injury [20]. The levels of total bile acids (TBA) in the serum and liver were measured using the kit from Wuhan Huamei Bioengineering Institute.

### 2.6. Measurement of Serum Biochemical Indexes

Serum was prepared via centrifugation at 3,000 × g for 10 min according to the above method and used for ELISA measurement. The serum levels of tumor necrosis factor *α* (TNF-*α*), interleukin- (IL-) 6, IL-10, superoxide dismutase (SOD), and malondialdehyde (MDA) were measured by using corresponding ELISA kits (a detection range of 2 to 250 pg/mL for TNF-*α*, 1 to 200 pg/mL for IL-6, 5 to 400 pg/mL for IL-10, 0.78-50 ng/mL for SOD, and 30-2000 ng/mL) from Wanleibio.

### 2.7. Histological Analysis of the Pancreas and Liver Tissues

The pancreas and liver tissues were extracted at 6 h, 12 h, and 24 h after the establishment of the SAP-induced hepatocellular injury model. The tissues were fixed in 5% paraformaldehyde and embedded in paraffin. The embedded tissues were cut into 4 *μ*m slices, respectively, for staining with hematoxylin and eosin (H&E). The severity of liver tissue injury was evaluated by using the formula pathological score = edema + number of inflammatory cells + number of necrotic cells + bleeding. The severity of liver tissue injury was evaluated by using the formula pathological score = neutrophil infiltration and edema + swelling of liver cells and stenosis of liver sinusoids + eosinophilic changes/eosinophilic necrosis of liver cells + focal necrosis + hepatocytes having dual nuclei + Kupffer cell hyperplasia and hypertrophy [21]. Five different fields were examined in each group.

### 2.8. Immunohistochemistry Analysis

Immunohistochemistry analysis was performed to assess the in situ expressed level of apoptotic factors (BAX and cleaved caspase 3), JAK2, p-JAK2, STAT3, and p-STAT3 in hepatocellular tissues. The embedded slices were deparaffinized and treated with hydrogen peroxide for 10 min. Primary antibodies were added and incubated 10 h at 4°C. Biotin-labeled goat anti-rabbit IgG secondary antibodies (1 : 1000) were used and incubated at 37°C for 15 min. The slides received further incubation at 37°C for 15 min with peroxidase-conjugated streptavidin. The section was stained with 3,3′-diaminobenzidine (DAB) and counterstained with hematoxylin. The color departure was performed using 2% hydrochloride and alcohol and 10 min splashing. The expression levels of target protein were analyzed using ImageJ software. According to a previous report [22], IHC scores were measured using the following standard: score 1, weak staining in <50% or moderate staining in <20% of all cells; score 2, weak staining in ≧50%, moderate staining in 20-50% or strong staining in <20% of all cells; and score 3, moderate staining in ≧50% or strong staining in ≧20% of all cells.

### 2.9. Reverse Transcription-Quantitative PCR (RT-qPCR)

RNA was isolated from 10-mg hepatocellular tissues using TRIzol reagent. cDNA was synthesized using a reverse transcription kit. The following primers were used: forward primer 5′-GGGACGAACTGGACAGTAACAT-3′ and reverse primer 5′-GGAGTCTCACCCAACCACCCT-3′ [23]; caspase 3, forward primer 5′-ACGGTACGCGAAGAAA-AGTGAC-3′ and reverse primer 5′-TCCTGACTTCGTATTTCAGGGC-3′ [24]; JAK2 forward, primer 5′-GCAGCCCTAAGGACTTCAAC-3′ and reverse primer 5′-CCGCTGAGGTTGTATTCTCC-3′ [25]; STAT3, forward primer 5′-TGGAAGAGGCGGCAGCAGATAGC-3′ and reverse primer 5′-CACGGCCCCCATTCCCACAT-3′ [26]; and *β*-actin, forward primer 5′-TTGCTGATCCACATCTGCTG-3′ and reverse primer 5′-GACAGGATGCAGAAGGAGAT-3′ [27]. Relative mRNA levels were standardized to *β*-actin via a *ΔΔ*Ct method.

### 2.10. Western Blot

10 mg hepatocellular tissues were pulverized in liquid nitrogen, and protein was obtained using RIPA lysis. Protein concentration quantified using the BCA kit. About 5.32-9.55 *μ*g of protein in 20 *μ*L for each sample was separated using SDS-PAGE and moved to the PVDF membrane. The membrane was blocked for 1 h at 22°C in 5% nonfat milk and probed with primary antibodies for 4 h at 37°C, rinsed four times with PBTB, incubated with secondary antibody for 2 h at 37°C, and washed in PBS. Target protein bands were obtained on Gel image processing system (Beijing Liuyi). Relative protein levels were evaluated using Gel-Pro-Analyzer and *β*-actin.

### 2.11. Terminal Deoxynucleotidyl Transferase (TdT) dUTP Nick-End Labeling (TUNEL) Assay

4 *μ*m sections of tissues were exposed to an apoptosis-specific staining kit (TUNEL assay (Wanleibio, Shenyang, China) based on the manufacturer's instructions. Quantitative analysis was carried out by measuring TUNEL-positive (apoptotic) cells, which were quantified by counting amber-colored cells in five fields.

### 2.12. Statistical Analyses

Data are presented as the means ± standard error of the mean (SEM) and analyzed using the SPSS 21.0 software (SPSS, Inc., Chicago, IL, USA). Student's *t*-test and one-way analysis of variance (ANOVA) with post hoc Tukey's tests were used to evaluate the variables between groups. Paeoniflorin and baicalin occupy the main proportion of Picroside II, and the Pearson correlation coefficient test was used to explore the relationship between serum levels and oxidative and/or inflammatory factors. The statistical difference was significant if the value of *P* < 0.05.

## 3. Results

### 3.1. Picroside II Treatment Reduced the Levels of Liver Enzymes

**Figure 1:**
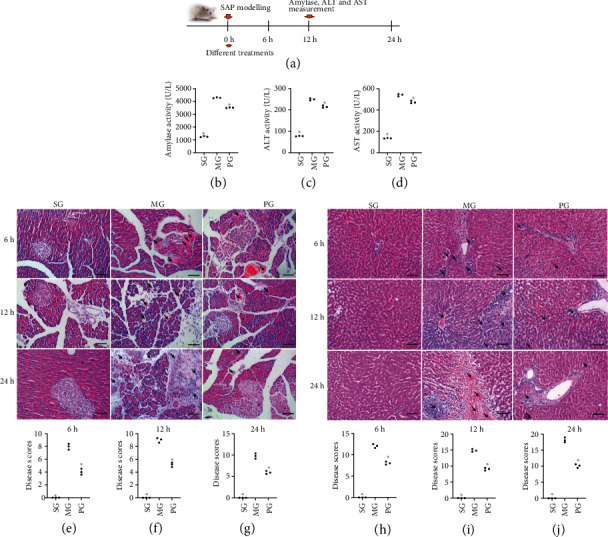
The effects of Picroside II on the liver injury. (a) The time flowchart on the biochemical analysis. (b) Amylase (AMY). (c) ALT. (d) AST. (e) Hematoxylin and Eosin (H&E) staining analysis of the effects of Picroside II on serious acute pancreatitis (SAP) and disease scores at 6 hours. (f) Disease scores at 12 hours. (g) Disease scores at 24 hours. (h) Hematoxylin and Eosin (H&E) staining analysis of the effects of Picroside II on serious acute pancreatitis- (SAP-) induced hepatocellular injury. Disease scores at 6 hours. (i) Disease scores at 12 hours. (j) Disease scores at 24 hours. All rats were divided into 3 groups, the sham group (SG), the model group (MG), and the Picroside II group (PG). The arrows show the inflammatory cells. *n* = 3 for each group. Scale bar = 30 *μ*m. ^∗^*P* < 0.05 vs. the MG group.

AMY activity increased in the MG group when compared with the SG group while Picroside II treatment reduced AMY activity (Figure 1(b), *P* < 0.05). Similarly, ALT (Figure 1(c)) and AST (Figure 1(d)) activity increased in the MG group when compared with the SG group while Picroside II treatment reduced ALT and AST activities (*P* < 0.05). AMY, ALT, and AST can help to identify hepatocellular injury, and the present results suggest that SAP induces hepatocellular injury by increasing the activities of AMY, ALT, and AST. Picroside II shows protective functions for hepatocellular injury by reducing the activities of AMY, ALT, and AST in the SAP-induced hepatocellular injury model.

### 3.2. Picroside II Treatment Reduced the Severity of SAP

The establishment of the SAP caused edema, the appearance of inflammatory cells, necrotic cells, and bleeding in pancreatitis tissues at 6 hours (Figure 1(e)), 12 hours (Figure 1(f)), and 24 hours (Figure 1(g), *P* < 0.05) when compared with those in the SG group. By contrast, Picroside II treatment reduced the severity of SAP at 6 hours (Figure 1(e)), 12 hours (Figure 1(f)), and 24 hours (Figure 1(g), *P* < 0.05) when compared with those in the MG group. The results suggest that Picroside II treatment reduces the severity of SAP.

### 3.3. Picroside II Treatment Reduced SAP-Induced Hepatocellular Injury

The establishment of the SAP-induced hepatocellular injury model increased neutrophil infiltration and edema, swelling of liver cells and stenosis of liver sinusoids, eosinophilic changes/eosinophilic necrosis of liver cells, focal necrosis, dual nuclei in hepatocytes, Kupffer cell hyperplasia, and hypertrophy at 6 hours (Figure 1(h)), 12 hours (Figure 1(i)), and 24 hours (Figure 1(j), *P* < 0.05) when compared with those in the SG group. By contrast, Picroside II treatment reduced the SAP-induced hepatocellular injury at 6 hours (Figure 1(h)), 12 hours (Figure 1(i)), and 24 hours (Figure 1(j), *P* < 0.05) when compared with those in the MG group. The results suggest that Picroside II treatment reduces SAP-induced hepatocellular injury.

### 3.4. SAP Did Not Induce Cholestasis

**Figure 2:**
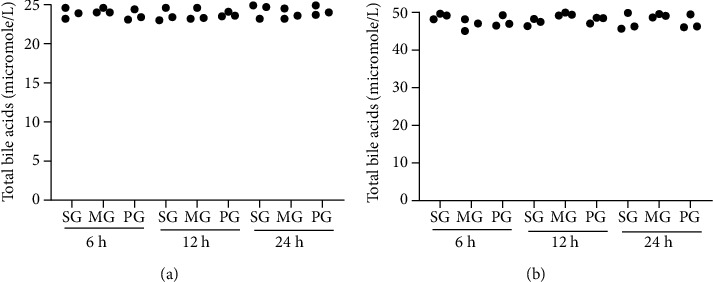
The total bile acid (TBA) levels. (a) Serum levels of TBA among different groups. (b) TBA levels in the liver among different groups. *n* = 3 for each group.

The results showed that the statistical differences for TBA levels in the serum and liver were insignificant (Figures 2(a) and 2(b)), suggesting that the SAP model did not induce cholestasis.

### 3.5. Picroside II Treatment Increased Antioxidant Properties in the SAP-Induced Hepatocellular Injury Model

**Figure 3:**
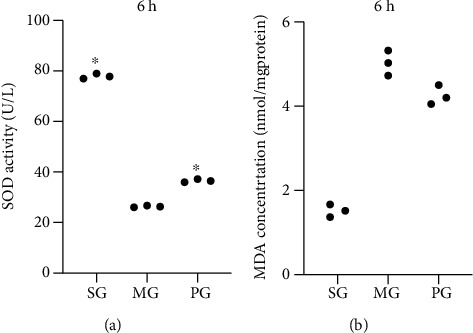
The effects of Picroside II on the levels of oxidative stress markers. (a) Superoxide dismutase (SOD) activity at 6 hours. (b) Malondialdehyde (MDA) concentration at 6 hours. ^∗^*P* < 0.05 vs. the MG group. *n* = 3 for each group.

SOD activity is commonly known as a marker of antioxidant properties. After the establishment of the SAP-induced hepatocellular injury model, the activities of SOD reduced at 6 hours (Figure 3(a)), 12 hours (Fig. S1A), and 24 hours (Supporting material; Fig. S1B, *P* < 0.05) when compared with those in the SG group. By contrast, Picroside II treatment increased the activities of SOD at 6 hours (Figure 3(a)), 12 hours (Fig. S1A), and 24 hours (Fig. S1B, *P* < 0.05) when compared with those in the MG group. The MDA level is commonly known as a marker of oxidative stress and its level increased at 6 hours (Figure 3(a)), 12 hours (Fig. S1C), and 24 hours (Fig. S1D, *P* < 0.05) when compared with those in the SG group. By contrast, Picroside II treatment reduced the concentration of MDA at 6 hours (Figure 3(a)), 12 hours (Fig. S1C), and 24 hours (Fig. S1D, *P* < 0.05) when compared with those in the MG group. The results suggest that Picroside II treatment increases antioxidant properties in the SAP-induced hepatocellular injury.

### 3.6. Picroside II Treatment Increased Anti-Inflammatory Properties in the SAP-Induced Hepatocellular Injury

**Figure 4:**
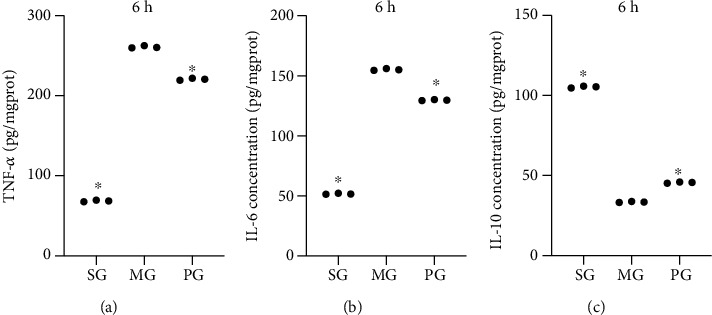
The effects of Picroside II on the serum levels of inflammatory cytokines. (a) Tumor nuclear factor- (TNF-) *α* level at 6 hours. (b) Interleukin-6 (IL-6) level at 6 hours. (c) Interleukin-10 (IL-10) level at 6 hours. ^∗^*P* < 0.05 vs. the MG group. *n* = 3 for each group.

TNF-*α* and IL-6 are widely reported proinflammatory cytokines [28], and IL-10 is a potent anti-inflammatory immunosuppressive cytokine [29]. After the establishment of the SAP-induced hepatocellular injury, the levels of TNF-*α* increased at 6 hours (Figure 4(a)), 12 hours (Fig. S2A), and 24 hours (Fig. S2B, *P* < 0.05) when compared with those in the SG group. By contrast, Picroside II treatment reduced the levels of TNF-*α* at 6 hours (Figure 4(a)), 12 hours (Fig. S2A), and 24 hours (Fig. S2B, *P* < 0.05) when compared with those in the MG group. After the establishment of the SAP-induced hepatocellular injury, the levels of IL-6 increased at 6 hours (Figure 4(b)), 12 hours (Fig. S2C), and 24 hours (Fig. S2D, *P* < 0.05) when compared with those in the SG group. By contrast, Picroside II treatment reduced the levels of IL-6 at 6 hours (Figure 4(b)), 12 hours (Fig. S2C), and 24 hours (Fig. S2E, *P* < 0.05) when compared with those in the MG group. By contrast, after the establishment of the SAP-induced hepatocellular injury, the levels of IL-10 reduced at 6 hours (Figure 4(c)), 12 hours (Fig. S2E), and 24 hours (Fig. S2F, *P* < 0.05) when compared with those in the SG group. By contrast, Picroside II treatment increased the levels of IL-10 at 6 hours (Figure 4(c)), 12 hours (Fig. S2E), and 24 hours (Fig. S2F, *P* < 0.05) when compared with those in the MG group. The results suggest that Picroside II treatment increased anti-inflammatory properties in the SAP-induced hepatocellular injury.

### 3.7. Picroside II Treatment Reduced Relative mRNA Levels of Apoptotic Factors and JAK2/STAT3 in Hepatocellular Tissues

**Figure 5:**
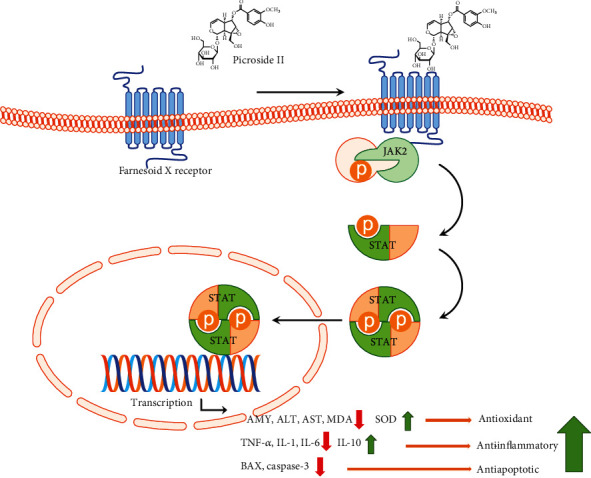
The functional mechanism of Picroside II. Picroside II may exert its function by affecting JAK2/STAT3 phosphorylation signaling, which results in the increase in antioxidant, anti-inflammatory, and antiapoptotic activities.

**Figure 6:**
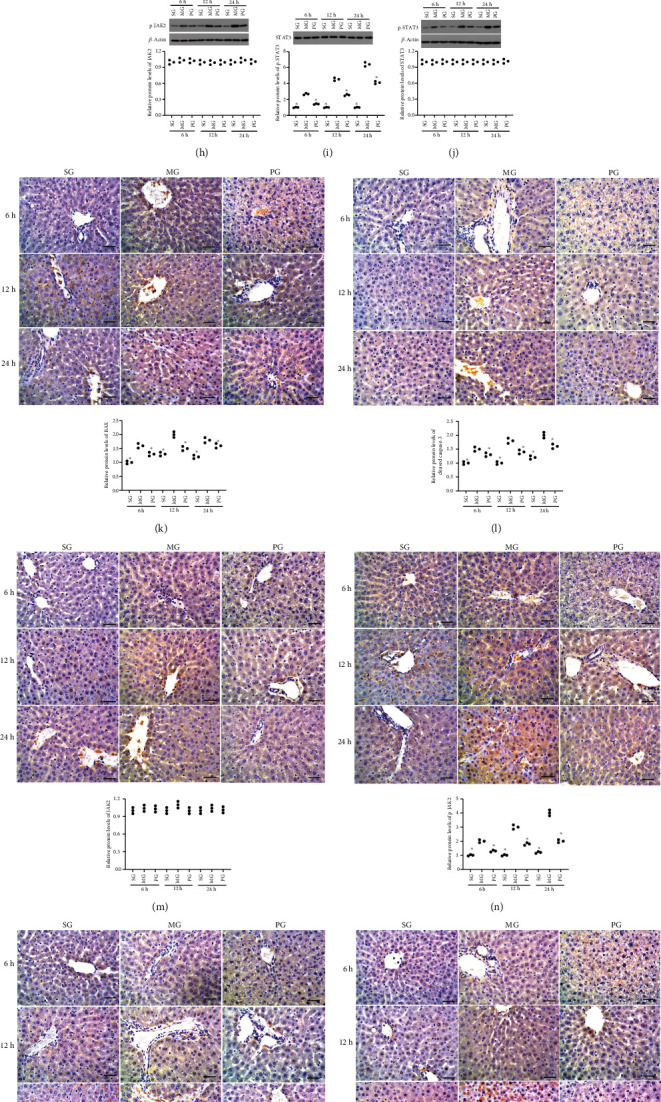
The effects of Picroside II on the levels of apoptotic factors and the Janus kinase 2 (JAK2)/signal transducer and activator of transcription 3 (STAT3) in hepatocellular tissues. (a) Relative mRNA levels of BAX. (b) Relative mRNA levels of caspase 3. (c) Relative mRNA levels of JAK2. (d) Relative mRNA levels of STAT3. (e) Relative protein levels of BAX. (f) Relative protein levels of caspase 3. (g) Relative protein levels of p-JAK2. (h) Relative protein levels of JAK2. (i) Relative protein levels of STAT3. (j) Relative protein levels of p-STAT3. (k) The expression of BAX. (l) The expression of caspase 3. (m) The expression of JAK2. (n) The expression of p-JAK2. (o) The expression of STAT3. (p) The expression of p-STAT3. Scale bar = 30 *μ*m. ^∗^*P* < 0.05 vs. the MG group. *n* = 3 for each group.

BAX and cleaved caspase 3 are widely reported apoptotic factors [30], and JAK2/STAT3 are potent pathway mediating inflammatory responses in the SAP-induced hepatocellular injury model [31]. For apoptotic factors, after the establishment of the SAP-induced hepatocellular injury model, relative mRNA levels of BAX increased at 6 hours, 12 hours, and 24 hours (Figure 5(a), *P* < 0.05) when compared with those in the SG group. By contrast, Picroside II treatment reduced relative mRNA levels of BAX at 6 hours, 12 hours, and 24 hours (Figure 6(a), *P* < 0.05) when compared with those in the MG group. After the establishment of the SAP-induced hepatocellular injury model, relative mRNA levels of cleaved caspase 3 increased at 6 hours, 12 hours, and 24 hours (Figure 6(b), *P* < 0.05) when compared with those in the SG group. By contrast, Picroside II treatment reduced relative mRNA levels of cleaved caspase 3 at 6 hours, 12 hours, and 24 hours (Figure 6(b), *P* < 0.05) when compared with those in the MG group. For JAK2/STAT3 signaling, after the establishment of the SAP-induced hepatocellular injury model, relative mRNA levels of JAK2 changed at 6 hours, 12 hours, and 24 hours (Figure 6(c), *P* > 0.05) when compared with those in the SG group. Picroside II treatment did not affect relative mRNA levels of JAK2 at 6 hours, 12 hours, and 24 hours (Figure 6(c), *P* > 0.05) when compared with those in the MG group. After the establishment of the SAP-induced hepatocellular injury model, relative mRNA levels of STAT3 changed less at 6 hours, 12 hours, and 24 hours (Figure 6(d), *P* > 0.05) when compared with those in the SG group. Picroside II treatment did not change relative mRNA levels of STAT3 at 6 hours, 12 hours, and 24 hours (Figure 6(d), *P* > 0.05) when compared with those in the MG group.

### 3.8. Picroside II Treatment Reduced Relative Protein Levels of Apoptotic Factors and JAK2/STAT3 in Hepatocellular Tissues

BAX and cleaved caspase 3 are widely reported apoptotic factors, and JAK2/STAT3 is a potent pathway mediating inflammatory responses in the SAP-induced hepatocellular injury model. For apoptotic factors, after the establishment of the SAP-induced hepatocellular injury model, relative protein levels of BAX increased at 6 hours, 12 hours, and 24 hours (Figure 6(e), *P* < 0.05) when compared with those in the SG group. By contrast, Picroside II treatment reduced relative protein levels of BAX at 6 hours, 12 hours, and 24 hours (Figure 6(e), *P* < 0.05) when compared with those in the MG group. After the establishment of the SAP-induced hepatocellular injury model, relative protein levels of cleaved caspase 3 increased at 6 hours, 12 hours, and 24 hours (Figure 6(f), *P* < 0.05) when compared with those in the SG group. By contrast, Picroside II treatment reduced relative protein levels of cleaved caspase 3 at 6 hours, 12 hours, and 24 hours (Figure 6(f), *P* < 0.05) when compared with those in the MG group. For JAK2/STAT3 signaling, after the establishment of the SAP-induced hepatocellular injury model, relative protein levels of JAK2 changed less at 6 hours, 12 hours, and 24 hours (Figure 6(g), *P* > 0.05) when compared with those in the SG group. Picroside II treatment did not change relative protein levels of JAK2 at 6 hours, 12 hours, and 24 hours (Figure 6(g), *P* > 0.05) when compared with those in the MG group. In contrast, after the establishment of the SAP-induced hepatocellular injury model, relative protein levels of p-JAK2 increased at 6 hours, 12 hours, and 24 hours (Figure 6(h), *P* < 0.05) when compared with those in the SG group. Picroside II treatment reduced relative protein levels of p-JAK2 at 6 hours, 12 hours, and 24 hours (Figure 6(h), *P* < 0.05) when compared with those in the MG group. After the establishment of the SAP-induced hepatocellular injury model, relative protein levels of STAT3 changed less at 6 hours, 12 hours, and 24 hours (Figure 6(i), *P* > 0.05) when compared with those in the SG group. Picroside II treatment did not change relative protein levels of STAT3 at 6 hours, 12 hours, and 24 hours (Figure 6(i), *P* > 0.05) when compared with those in the MG group. In contrast, after the establishment of the SAP-induced hepatocellular injury model, relative protein levels of p-STAT3 increased at 6 hours, 12 hours, and 24 hours (Figure 6(j), *P* < 0.05) when compared with those in the SG group. Picroside II treatment reduced relative protein levels of p-STAT3 at 6 hours, 12 hours, and 24 hours (Figure 6(j), *P* < 0.05) when compared with those in the MG group. The results suggest that Picroside II treatment increases antiapoptosis and anti-inflammatory activities by reducing relative protein levels of apoptotic factors and JAK2/STAT3 signaling in hepatocellular tissues.

### 3.9. Picroside II Treatment Reduced the Expression of Apoptotic Factors and JAK2/STAT3 in Hepatocellular Tissues

For apoptotic factors, after the establishment of the SAP-induced hepatocellular injury model, the expression of BAX increased at 6 hours, 12 hours, and 24 hours (Figure 6(k), *P* < 0.05) when compared with those in the SG group. By contrast, Picroside II treatment reduced the expression of BAX at 6 hours, 12 hours, and 24 hours (Figure 6(k), *P* < 0.05) when compared with those in the MG group. After the establishment of the SAP-induced hepatocellular injury model, the expression of cleaved caspase 3 increased at 6 hours, 12 hours, and 24 hours (Figure 6(l), *P* < 0.05) when compared with those in the SG group. By contrast, Picroside II treatment reduced the expression of cleaved caspase 3 at 6 hours, 12 hours, and 24 hours (Figure 6(l), *P* < 0.05) when compared with those in the MG group. For JAK2/STAT3 signaling, after the establishment of the SAP-induced hepatocellular injury model, the expression of JAK2 changed less at 6 hours, 12 hours, and 24 hours (Figure 6(m), *P* > 0.05) when compared with those in the SG group. Picroside II treatment did not change the expression of JAK2 at 6 hours, 12 hours, and 24 hours (Figure 6(m), *P* > 0.05) when compared with those in the MG group. In contrast, after the establishment of the SAP-induced hepatocellular injury model, the expression of p-JAK2 increased at 6 hours, 12 hours, and 24 hours (Figure 6(n), *P* < 0.05) when compared with those in the SG group. Picroside II treatment reduced the expression of p-JAK2 at 6 hours, 12 hours, and 24 hours (Figure 6(n), *P* < 0.05) when compared with those in the MG group. After the establishment of the SAP-induced hepatocellular injury model, the expression of STAT3 changed less at 6 hours, 12 hours, and 24 hours (Figure 6(o), *P* > 0.05) when compared with those in the SG group. Picroside II treatment did not change the expression of STAT3 at 6 hours, 12 hours, and 24 hours (Figure 6(o), *P* > 0.05) when compared with those in the MG group. In contrast, after the establishment of the SAP-induced hepatocellular injury model, the expression of p-STAT3 increased at 6 hours, 12 hours, and 24 hours (Figure 6(p), *P* < 0.05) when compared with those in the SG group. Picroside II treatment reduced the expression of p-STAT3 at 6 hours, 12 hours, and 24 hours (Figure 6(p), *P* < 0.05) when compared with those in the MG group. The results suggest that Picroside II treatment increases antiapoptosis and anti-inflammatory activities by reducing the expression of apoptotic factors and JAK2/STAT3 signaling in hepatocellular tissues.

### 3.10. Picroside II Treatment Reduced Hepatocellular Apoptosis

**Figure 7:**
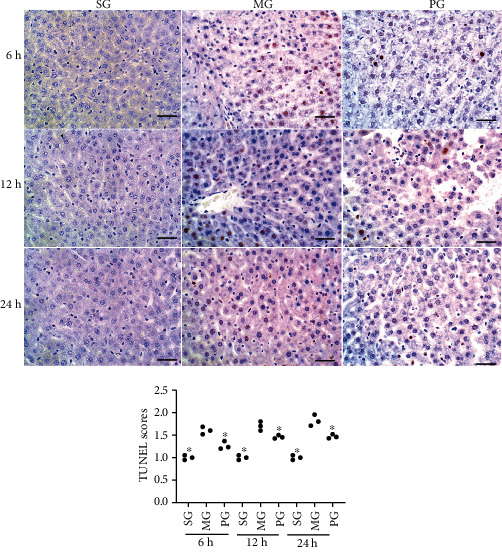
Terminal deoxynucleotidyl transferase dUTP nick end labeling (TUNEL) assay on the effects of Picroside II on the hepatocellular apoptosis (magnification 400x). Scale bar = 30 *μ*m. ^∗^*P* < 0.05 vs. the MG group. *n* = 3 for each group.

TUNEL analysis showed that the establishment of the SAP-induced hepatocellular injury model increased the severity of apoptosis of hepatocellular tissues by increasing TUNEL scores at 6 hours, 12 hours, and 24 hours (Figure 7, *P* < 0.05). By contrast, Picroside II treatment reduced *t*he apoptotic severity of hepatocellular tissue at 6 hours, 12 hours, and 24 hours (Figure 7, *P* < 0.05) when compared with those in the MG group. The results suggest that Picroside II treatment reduces the severity of hepatocellular apoptosis.

## 4. Discussion

In this study, an SAP-induced hepatocellular injury model was established using Pentobarbital sodium and confirmed in the pancreas tissues, including the appearance of edema, inflammatory cells, necrotic cells, and bleeding (Figure 1). Meanwhile, SAP-induced hepatocellular injury was detected from the following two aspects: (1) the activities of the enzymes (AMY, ALT, and AST) associated with hepatocellular injury increased (Figure 1), and (2) hepatocellular injury was further observed in the tissues, including neutrophil infiltration and edema, swelling of liver cells and stenosis of liver sinusoids, eosinophilic changes/eosinophilic necrosis of liver cells, focal necrosis, dual nuclei in one cell, Kupffer cell hyperplasia, and hypertrophy (Figure 1). All results suggest that the SAP-induced hepatocellular injury model was successfully established. The model establishment affected the levels of oxidative stress biomarkers (Figure 3) and increased the levels inflammatory factors (Figure 4), apoptotic factors and inflammatory signaling JAK2/STAT3 (Figure 6), and pancreas tissue apoptosis (Figure 7). Picroside II treatment repaired the hepatocellular injury caused by the SAP-induced hepatocellular injury model (Figure 1) by affecting the levels of oxidative stress biomarkers (Figure 3), inflammatory factors (Figure 4), apoptotic factors and inflammatory signaling JAK2/STAT3 (Figure 6), and tissue apoptosis (Figure 7).

SAP contributes to hepatocyte injury during acute pancreatitis, and SAP-induced hepatocellular injury is consistent with the previous reports [3]; the JAK2/STAT3 signaling pathway plays an important role on liver injury associated with SAP in the rat model. However, there is still partial difference with the report, which is not involved with the study on the changes of p-JAK2 and p-STAT3. Conversely, the present study did not show the changes of JAK2 and STAT3 for the SAP-induced hepatocellular injury model. Other mechanisms for SAP-induced hepatocellular injury also existed. For an example, pancreatitis-induced ascitic fluid (PAF) may cause liver injury and hepatocyte apoptosis by affecting p38-MAPK and cleaved caspase 3 [32].

Picroside II exerted protective function in the SAP-induced hepatocellular injury model. Picroside II treatment increased the levels of SOD and reduced the MAD level. The treatment resulted in the reduction in the levels of proinflammatory cytokines TNF-*α* and IL-6 and the increase in IL-10 level. Picroside II intervention reduced the levels of BAX and cleaved caspase 3. Therefore, Picroside II treatment increased antioxidant, anti-inflammatory, and antiapoptosis capacities in the model. Picroside II also had been reported to have inhibitory function on oxidative signaling pathway [11] and reduce inflammatory and apoptotic cells [33]. A previous wok also showed its antioxidant and anti-inflammatory properties in a kidney ischemia/reperfusion injury model by the affecting TLR4/NF-*κ*B signaling [34]. Present findings demonstrated that Picroside II showed significant inhibitory functions on the apoptosis of the model by affecting the levels of BAX, cleaved caspase 3 (Figure 6), and TUNEL scores of hepatocellular tissues (Figure 7), which is also consistent with a previous report that Picroside II had a protective function for hepatocyte and exerted an inhibitory impact on hepatocyte apoptosis [35].

The present findings show that Picroside II treatment increases antioxidant, anti-inflammatory, and antiapoptotic activities in the SAP model. The possible mechanisms may be explained as follows: Picroside II displays protective function for liver injury possibly through activation of farnesoid X receptor [36], which reduces oxidative, inflammatory, and apoptotic activities by affecting JAK2/STAT3 signaling (Figure 5) [37]. The levels of serum AMY, ALT, AST, IL-6, TNF-*α*, MDA, and SOD are closely associated with the SAP progression in the patients with liver function damage, and effective therapy can reduce the levels of AMY, ALT, AST, IL-6, TNF-*α*, and MDA and increase the levels of SOD. A previous study showed that the IL-10 polymorphism can affect SAP and IL-10-1082A allele exerts a protective factor in SAP patients [38]. Comparatively, the levels of p-JAK2, p-STAT3, BAX, and caspase 3 were widely reported in animal models and seldom reported in the study on the prognosis of the SAP human patients.

There were some limitations in the present study. Although Picroside II treatment changed the levels of apoptotic factors and inflammatory signaling JAK2/STAT3, the association between the signaling molecules and inflammatory cytokines, or oxidative biomarkers, and/or apoptotic factors was not explored in the present study. The exact mechanism for SAP-induced hepatocellular injury remained unclear. The exact association between the signaling molecules and inflammatory cytokines, or oxidative biomarkers, and-or apoptotic factors may be explored by using target gene overexpression or knockout in the animal models. The important issue will be considered in our future work. The direct function of Picroside II on oxidative stress was performed in this study, such as the effect of Picroside II on ROS contents. A further work is needed to be performed to address these issues in the future.

## Data Availability

The original data of the experiment can be obtained by email from the corresponding author.

## Supplementary Materials

Supplementary Materials Fig. S1: the effects of Picroside II on the levels of oxidative stress markers. A, SOD activity at 12 hours. B, SOD activity at 24 hours. C, MDA concentration at 12 hours. D, MDA concentration at 24 hours. ^∗^*P* < 0.05 vs. the MG group. *n* = 3 for each group. Fig. S2: the effects of Picroside II on the serum levels of inflammatory cytokines. A, TNF-*α* level at 12 hours. B, TNF-*α* level at 24 hours. C, IL-6 level at 12 hours. D, IL-6 level at 24 hours. E, IL-10 level at 12 hours. F, IL-10 level at 24 hours. ^∗^*P* < 0.05 vs. the MG group. *n* = 3 for each group.
