# Supplementary material for: Picroside II Improves Severe Acute Pancreatitis-Induced Hepatocellular Injury in Rats by Affecting JAK2/STAT3 Phosphorylation Signaling
Source: Biomed Res Int. 2021 Jul 27;2021:9945149. doi: 10.1155/2021/9945149 (PMC8337114; doi:10.1155/2021/9945149)
Supplement: Supplementary Materials — Fig. S1: the effects of Picroside II on the levels of oxidative stress markers. A, SOD activity at 12 hours. B, SOD activity at 24 hours. C, MDA concentration at 12 hours. D, MDA concentration at 24 hours. ∗P < 0.05 vs. the MG group. n = 3 for each group. Fig. S2: the effects of Picroside II on the serum levels of inflammatory cytokines. A, TNF-α level at 12 hours. B, TNF-α level at 24 hours. C, IL-6 level at 12 hours. D, IL-6 level at 24 hours. E, IL-10 level at 12 hours. F, IL-10 level at 24 hours. ∗P < 0.05 vs. the MG group. n = 3 for each group. [file 9945149.f1.doc]

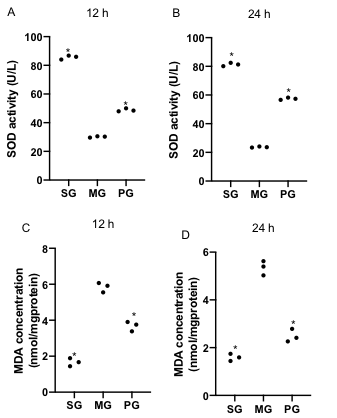


Fig. S1. The effects of Picroside II on the levels of oxidative stress markers. A, SOD activity at 12 hours. B, SOD activity at 24 hours. C, MDA concentration at 12 hours. D, MDA concentration at 24 hours. *P < 0.05 vs the MG group. n = 3 for each group.


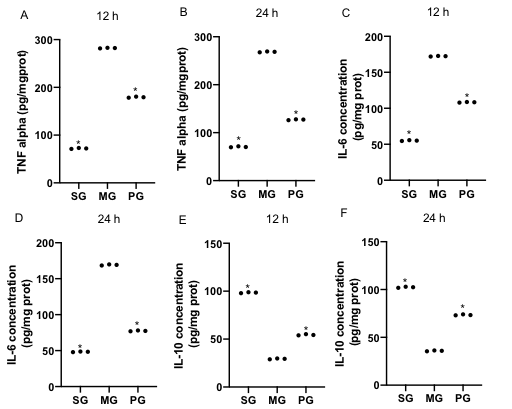


Fig. S2. The effects of Picroside II on the serum levels of inflammatory cytokines. A TNF-α level at 12 hours. B, TNF 𝝰 level at 24 hours. C, IL-6 level at 12 hours. D, IL-6 level at 24 hours. E, IL-10 level at 12 hours. F, IL-10 level at 24 hours. *P < 0.05 vs the MG group. n = 3 for each group.
